# Severe COVID-19 multisystem inflammatory syndrome versus severe dengue in children from Indonesia: a cross-sectional study

**DOI:** 10.1186/s12245-024-00658-6

**Published:** 2024-07-11

**Authors:** Anggraini Alam, Fina Meilyana Andriyani, Stanza Uga Peryoga

**Affiliations:** 1 Infection & Tropical Diseases Division, Department of Child Health, Faculty of Medicine, Universitas Padjadjaran-Hasan Sadikin General Hospital, Bandung, Indonesia; 2 Emergency and Intensive Care Division, Department of Child Health, Faculty of Medicine, Universitas Padjadjaran-Hasan Sadikin General Hospital, Bandung, Indonesia

**Keywords:** Severe MIS-C, Severe dengue, Clinical manifestations, Laboratory parameters

## Abstract

**Introduction:**

Severe multisystem inflammatory syndrome in children (MIS-C) and severe dengue are challenging to identify during the COVID-19 pandemic in dengue-endemic areas. Fever, multiorgan involvement, and shock characterize both severe MIS-C and severe dengue. Distinguishing between the two diseases is beneficial in initiating proper management.

**Methods:**

Medical records of children < 18 years old who were hospitalized at Hasan Sadikin General Hospital’s PICU between December 2020 and July 2022 with severe MIS-C or severe dengue were recorded. Differences were assessed using comparative and descriptive analyses.

**Results:**

Seventeen severe dengue patients and 4 severe MIS-C were included. The average age of severe MIS-C was 11.5 years (SD ± 2.9, 95% CI), and that of severe dengue patients was 6.2 years (SD ± 4.4, 95% CI) (p value = 0.034, 95%). Fever and abdominal pain were the most common symptoms in both groups (*p* = 0.471, 95% CI). Rash (*p* = 0.049) and nonpurulent conjunctivitis (*p* = 0.035) were two symptoms with significant differences. The highest platelet count (p-value = 0.006, 95% CI), AST (p-value = 0.026, 95% CI), and D-dimer level (p-value = 0.025, 95% CI) were significantly different between the two cohorts. Cardiac abnormalities were found in all (100%) severe MIS-C patients, but only one (5.9%) in severe dengue patients.

**Conclusion:**

Age, rash, nonpurulent conjunctivitis, platelet count, AST and D-dimer level may distinguish severe MIS-C from severe dengue fever.

## Introduction

The COVID-19 pandemic has led to medical breakthroughs. One of them is the emergence of post-COVID-19 infection, MIS-C in children or paediatric inflammatory multisystem syndrome temporally associated with SARS-CoV-2 infection (PIMS-TS) in Europe. This phenomenon requires hospitalization and even admission to the intensive care unit (ICU). Approximately 68–88% of previously healthy children with MIS-C required treatment in a paediatric ICU (PICU), with 45% admitted the same day as hospitalization, 63–64% requiring inotropic support, and 20–28% requiring mechanical breathing. [1–6].

Initially, MIS-C was identified as an illness similar to Kawasaki disease (KD) or toxic shock syndrome (TSS). This condition occurs in individuals who has previous contact or are diagnosed with COVID-19 and who experience symptoms related to their gastrointestinal, nervous, and myocardial symptoms, occasionally leading to shock [7–11]. The World Health Organization (WHO), U.S. Centers for Disease Control and Prevention (CDC), and the United Kingdom Royal College of Paediatrics and Child Health (RCPCH) have developed diagnostic criteria for MIS-C or PIMS-TS [12–14].

However, as a dengue-endemic country, Indonesia also faces certain problems distinguishing severe MIS-C from severe dengue infection. Dengue itself also presents with fever, abdominal pain, and multiple organ involvement. When severe dengue develops, severe plasma leakage can lead to shock, and endothelial dysfunction and alterations in vascular permeability can potentially contribute to cardiovascular manifestations [15, 16]. The emergency management of dengue is crucial because 25% of children progress to severe dengue, and the mortality rate reaches 20% if left untreated [17, 18]. Given the many similarities among both diseases, evaluating clinical symptoms and laboratory findings is beneficial for diagnosis and management. This study presents cases of severe MIS-C compared to severe dengue and overlapping diagnoses between MIS-C and severe dengue in children admitted to the PICU of Hasan Sadikin General Hospital, Bandung.

## Materials and methods

### Study population

This cross-sectional study involved the use of retrospective data from all children < 18 years old who were admitted to the PICU of Hasan Sadikin General Hospital, Bandung, Indonesia, between December 2020 and July 2022, and who met the criteria for severe MIS-C and severe dengue.

### Case definition

The WHO case definition was used to diagnose MIS-C and was determined by paediatric professionals. According to the WHO, MIS-C should be evaluated in children aged 0 to 19 years who have a fever for ≥3 days, followed by clinical signs of multisystem involvement, elevated markers of inflammation, no other apparent microbial cause of inflammation, and evidence of SARS-CoV-2 infection [19]. According to the National Health Service (NHS) England consenses, severe MIS-C may be indicated by the following: (1) physiological features of severe disease: extended capillary refill time (CRT), persistent hypotension, persistent tachycardia, 40 mL/kg fluid bolus requirement, and oxygen saturation < 92% in room air; (2) haematological and biochemical features: clinically significant increases in CRP, troponin; NT-proBNP, lactate, ferritin, D-dimer, or lactate dehydrogenase (LDH), high or low fibrinogen, and increased creatinine, and (3) abnormal electrocardiogram results, coronary artery aneurysms on echocardiogram, or left ventricular failure [20].

Severe dengue was diagnosed using the 2009 WHO Dengue guidelines. Severe dengue involves severe plasma leakage as evidenced by a rising haematocrit and circulatory compromise that leads to shock or fluid accumulation; severe haemorrhage; or severe organ impairment, such as acute liver failure, acute renal failure, encephalopathy, or cardiomyopathy are examples of severe organ impairment [15].

### Data analysis

The patients’ clinical manifestations were recorded. Laboratory findings such as routine blood count (Mindray, China), alanine transaminase and aspartate transaminase (ALT and AST) (Mindray, China), blood urea and creatinine (Hitachi, Japan), D-dimer (Siemens Healthcare, Germany) or prothrombin time (PT), activated partial thromboplastin time (APTT), and international normalized ratio (INR) for coagulation markers (Sysmex Corp, Japan), C-reactive protein (CRP) or procalcitonin for inflammation markers (Mindray, China), and SARS-CoV-2 real-time polymerase chain reaction (RT-PCR), antigen, or antibody (Abbott, Panbio Ltd, USA). Non-structural protein 1 (NS1) and IgM/IgG antibody (Abbott Bioline, Panbio Ltd, USA) against dengue were also recorded. An ultrasonic cardiac output monitor (USCOM Ltd, Australia) was performed on all patients.

Statistical analysis will compare patient demographics, clinical symptoms, and laboratory profiles. Independent t tests were used to compare the means and standard deviations for continuous data. Nonparametric tests were used to calculated the median and interquartile range effect size if the data were not normally distributed. Categorical variables were tested with chi-square test. All statistical analyses used a p value of 0.05 and a 95% confidence interval. The statistical analysis was performed with SPSS 26.0 (SPSS, Chicago, USA).

## Results

Twenty-three children participated in the study. Five children had clinical symptoms of severe MIS-C, 17 had severe dengue, and one had both diseases. One child had a positive blood culture for *Staphylococcus aureus*, whereas the others did not. Thus, only one patient was excluded from the study, leaving four severe MIS-C patients, 17 severe dengue patients, and one patient with both diseases.

Two MIS-C patients were positive for SARS-CoV-2 IgG antibodies, and one had a positive RT-PCR result. The remaining two children tested negative for SARS-CoV-2 IgM and IgG. The anamnesis showed that both children had been in contact with their COVID-19-positive relatives one month prior to admission. Ten children in the severe dengue cohort tested positive for NS1 dengue antigen, four for IgM dengue antibody, two for IgG dengue antibody, and one for both IgM and IgG antibodies.

### Clinical manifestation of severe MIS-C and severe dengue patients

In the severe dengue cohort, the majority of individuals were male (64.7%). Conversely, in the severe MIS-C cohort, 75% of patients were female. Statistical analysis revealed no significant difference according to sex (p value = 0.149, 95% CI). On the other hand, the average age of children diagnosed with severe MIS-C was 11.5 years (SD ± 2.9, 95% CI), and was younger for severe dengue at 6.2 years old (SD ± 4.4, 95% CI), which was a significant difference (p value of 0.034, 95% CI).

All patients had a fever. Nonpurulent conjunctivitis was found in 25% of severe MIS-C patients. Conversely, 11 (64.7%) and 7 (41.2%) severe dengue patients had vomiting and diarrhoea, respectively. Clinical manifestations of rash (p value = 0.049, 95% CI) and nonpurulent conjunctivitis were significantly different between the two groups (p-value = 0.035, 95% CI). Two (50%) and eight (47.1%) patients with severe MIS-C and dengue, respectively, exhibited signs of fluid overload. Atypical haemorrhaging was observed in four patients (23.5%) with severe dengue. Additionally, hepatomegaly was present in three (17.6%) patients with severe dengue, and ascites was observed in two (11.8%) patients. In the MIS-C cohort, three patients (75%) displayed signs of ascites, and one (25%) experienced hepatomegaly. During hospitalization, more than half (58.8%) of the severe dengue patients did not require oxygen supplementation. The statistical analysis did not reveal any significant difference in oxygen need (p value = 0.122, 95% CI). Table 1 shows the clinical characteristics of the included patients.

**Table 1:** Clinical manifestations of children diagnosed with MIS-C and severe dengue

Characteristics	MIS-C (*n* = 4)	Severe Dengue (*n* = 17)	*p*-value*
**Gender** *– n (%)*			0.149
Male	1 (25.0)	11 (64.7)	
Female	3 (75.0)	6 (35.3)	
**Age** *– mean ± SD*	11.5 (2.9)	6.2 (4.4)	**0.034**
**Clinical manifestations** *– n (%)*			
Fever	4 (100.0)	17 (100.0)	n/a
Abdominal pain	4 (100.0)	15 (88.2)	0.619
Diarrhea	3 (75.0)	7 (41.2)	0.149
Vomiting	2 (50.0)	11 (64.7)	0.586
Rash	3 (75.0)	4 (23.5)	**0.049**
Non purulent conjunctivitis	1 (25.0)	0 (0.0)	**0.035**
Dizziness	2 (50.0)	3 (17.6)	0.172
**Oxygen requirements** *– n (%)*			0.122
No oxygen	1 (25.0)	10 (58.8)	
Nasal cannula	2 (50.0)	6 (35.3)	
Simple Mask	0 (0.0)	1 (5.9)	
Non-Rebreather	0 (0.0)	0 (0.0)	
Mechanical ventilation	1 (25.0)	0 (0.0)	

MIS-C, Multisystem Inflammatory Syndrome in Children; SD, standard deviation *Chi-square test for proportions, Independent t-test for comparing means

The Glasgow Coma Scale (GCS) showed that only two (17.8%) of the 17 severe dengue patients experienced a loss of consciousness. The GCS is a tool for objectively describing the extent of impaired consciousness, which consists of three parameters: eye response (E), verbal response (V), and motor response (M) [21]. The remaining 88.2% of severe dengue patients had a GCS score of 15. The median severe dengue GCS score was 15 (IQR 0, 95% CI), while for severe MIS-C the median was 15 (IQR 4.5, 95% CI), with one patient (25%) experienced loss of consciousness. There was no difference in the median values and other vital signs measured between patients with MIS-C and those with severe dengue.

The pulse pressure in severe dengue patients was narrower, with a median of 33.2 (IQR±11.1, 95% CI), than that in MIS-C patients (37.5 IQR±26.3, 95% CI). The median values for other critical vital signs, namely heart rate, respiration rate, temperature, and oxygen saturation, exhibited no significant variation. Table 2 displays the essential physiological indicators of children diagnosed with MIS-C and severe dengue.

**Table 2:** The difference of vital signs between MIS-C and severe dengue children

Characteristics	MIS-C (*n* = 4)	Severe Dengue (*n* = 17)	*p*-value*
**Vital Signs**			
GCS *– median ± IQR*	15.0 (4.5)	15.0 (0.0)	0.419
Systolic blood pressure *– median ± IQR*	120.0 (30.0)	100.0 (18.0)	0.080
Pulse pressure *– mean ± SD*	37.5 (26.3)	33.2 (11.1)	0.625
Heart rate *– median ± IQR*	120.0 (22.0)	110 (20.0)	0.476
Respiratory rate *– median ± IQR*	24.0 (0.0)	24.0 (7.0)	0.824
Temperature *– mean± SD*	37.8 (1.3)	37.2 (0.8)	0.223
Oxygen saturation *– median ± IQR*	98.0 (4.0)	97.0 (4.0)	0.471

MIS-C, Multisystem Inflammatory Syndrome in Children; GCS, Glasgow Coma Scale; SD, standard deviation; IQR, interquartile range * Independent t-test for parametric test and Mann-Whitney test for non-parametric test

### Laboratory findings of MIS-C and severe dengue patient

The highest platelet counts (p-value = 0.006, 95% CI), AST levels (p-value = 0.026, 95% CI), and D-Dimer levels (p-value = 0.025, 95% CI) were significantly different between the two groups. The mean and median values of these parameters were greater in MIS-C patients (highest mean platetet count 342,000, SD±230,262; median AST 32.0, IQR±178.7; median D-Dimer 3360, IQR±1761.8; 95%CI) than in severe dengue patients (highest mean platelet count 120,294, SD±100,767; median AST 301.0, IQR±570.3; median D-dimer 2.9, IQR±3.5; 95% CI). Table 3 displays the differences in the laboratory parameters between the two groups.

**Table 3:** The differences of laboratory parameters between MIS-C and severe dengue children

Laboratory Parameters	MIS-C	Severe Dengue	*p*-value**
Hemoglobin			
Highest*– mean ± SD*	11.5 (0.5)	14.1 (3.5)^#^	0.161
Lowest*– mean ± SD*	8.8 (0.8)*	9.7 (3.4)	0.729
Hematocrit			
Highest*– mean ± SD*	35.2 (1.7)	42.9 (9.4)^#^	0.123
Lowest*– mean ± SD*	28.4 (3.6)*	28.7 (9.1)	0.954
Leukocyte			
Highest*– mean ± SD*	14,395 (6876)	10,632 (5375)^#^	0.245
Lowest*– median ± IQR*	7070 (3510)*	4550 (12,570)	0.482
Platelet			
Highest*– mean ± SD*	342,000 (230,262)	120,294 (100,767)	**0.006**
Lowest*– median ± IQR*	109,000 (97,000)*	19,500 (264,000)	0.574
AST*– median ± IQR*	32.0 (178.7)	301.0 (570.3)^^^	**0.026**
ALT*– median ± IQR*	29.0 (101.3)	91.0 (324.0)*	0.161
Urea*– mean ± SD*	59.7 (27.4)	39.2 (21.9)^^^	0.178
Creatinine*– mean ± SD*	0.7 (0.3)	0.5 (0.2)^^^	0.179
D-Dimer*– median ± IQR*	3360 (1761.8)^#^	2.9 (3.5)^*Δ*^	**0.025**

*MIS-C: multisystem inflammatory syndrome in children; ALT: alanine transaminase; AST: and aspartate transaminase*

#missing data in 1 patient; *missing data in two patients; ^ missing data in three patients; Δdata from five patients. ** Mann-Whitney U test

Only half of MIS-C patients had increased AST and ALT, but 70.6% of severe dengue patients did. Among the children in the severe dengue category, three (17.6%) exhibited increased AST but normal ALT, while nine (52.9%) had elevated levels of both AST and ALT. Among the children with severe MIS-C, only two (50%) exhibited increased levels of both AST and ALT, while the others had normal levels of AST and ALT. The difference in AST and ALT levels between the two groups was statistically significant (median 301.0, IQR ± 570.3, p-value = 0.026; 95% CI). Albumin levels decreased in 58.82% of children with severe dengue and in 50% in MIS-C. Three children with MIS-C had drastically high D-dimer levels, while four of five children with severe dengue children had modestly elevated D-dimer results.

Cardiac output monitoring using ultrasound revealed cardiac abnormalities in all (100%) MIS-C patients. One severe MIS-C patient showed dilatation of the coronary artery, two patients (50%) experienced myocarditis with abnormal findings on electrocardiography, and the remaining patient had pericardial effusion along with bilateral pleural effusion. Moreover, echocardiography revealed reduced circulation, preload, contractility, and perfusion in one (5.9%) severe dengue patient. However, the Troponin I level of these patients were still within the normal range.

### Management of MIS-C and severe dengue in children

Four (23.5%) severe dengue patients developed progressive shock, and two of them, who were previously mentioned, experienced a decline in consciousness. All four patients required crystalloid and colloid resuscitation, and one patient needed continuous vasopressor infusion. Conversely, in the severe MIS-C cohort, two patients (50%) experienced shock. Both patients received crystalloid resuscitation, as well as inotropic and vasopressor medications.

Eight (47.1%) children with severe dengue were administered antibiotics, and two of these children (11.8%) were also administered steroids. Within the MIS-C cohort, a single patient (25%) with acute COVID-19 infection was administered remdesivir. The remaining patients in the cohort were administered antibiotics. All severe MIS-C patients received steroids, but only one received intravenous immunoglobulin (IVIG). Due to high D-dimer levels, two children received heparin. Children with severe MIS-C were given oral steroids and acetylsalicylic acid after discharge until their follow-up.

### A case of MIS-C and severe dengue overlap

One patient was diagnosed with both severe MIS-C and severe dengue. The patient was a 5-year-old male with a fever lasting for four days, accompanied by abdominal pain, nausea, vomiting, and dizziness. The mother acknowledged that her brother had contracted COVID-19 two weeks earlier and had finished self-isolation. Upon registration, the patient exhibited ascites and hepatomegaly, but his vital signs were stable. A bulla was found in his right lower extremity, along with petechiae and purpura on both lower extremities.

The dengue NS1 test was positive, while the SARS-CoV-2 antigen test was negative. The maximum recorded haemoglobin level was 17.2 gr/dL; its lowest was 3.8 gr/dL. A parallel pattern was found within haematocrit levels, ranging from a peak of 49.30% to a nadir of 11.0%. The leukocyte count reached a maximum of 17,070 cells/µL and a minimum of 5740 cells/µL. During hospitalization, the platelet count reached the lowest level of 10,000 cells/µL and the highest of 25,000 cells/µL. ALT and AST were increased, and urea and creatinine were modestly elevated. D-dimer, PT, aPTT, and the INR increased, but Troponin I barely elevated. A decrease in the serum albumin concentration was observed. Dual antibiotics, steroids, and IVIG were given.

## Discussion

This study compared the signs, clinical manifestations, and laboratory parameters of severe MIS-C and severe dengue PICU patients. The average age of children with severe MIS-C was 11.5 years (SD ± 2.9, 95% CI), which is in line with previous studies in which the average age of MIS-C were 6 to 12 years old [22, 23]. Conversely, 60% of primary dengue cases usually develop between 11 and 18 years of age. The likelihood of contracting primary or secondary dengue has been observed to increase with age in children [24–26]. Our analysis indicated that severe dengue typically develops at an average age of 6.2 years old (SD ± 4.4, 95% CI), consistent with previous studies [27–29].

Prior SARS-CoV-2 infection was documented in 58% of MIS-C patients [6], consistent with our study in which 50% of the MIS-C cohort was positive for SARS-CoV-2 antibody while the rest had contact with COVID-19 positive patients. Individuals with MIS-C develop postinfectious immunological dysregulation of the innate immune system, including activation of IL-1β and elevated levels of proinflammatory cytokines [28].

Nausea, vomiting, and diarrhoea have been documented in 2–20% of MIS-C patients, while abdominal discomfort only occurs in only 2% of individuals [30, 31]. Haemorrhagic, nonpurulent conjunctivitis affect 40–56% of MIS-C treated children [32]. In this study, fever and abdominal pain were the most common symptoms in both severe MIS-C and severe dengue patients. Nonpurulent conjunctivitis was observed only in the MIS-C cohort (*n* = 25%), which was confirmed by comparative analysis (p-value 0.035, 95% CI) and prior studies [32].

This study also revealed a lower highest platelet count, which was greater in the severe dengue cohort than in the severe MIS-C cohort, and exhibits significant variations (p value = 0.006; 95% CI). Dengue virus (DENV) can affect bone marrow progenitor cells by inducing hypoplasia during the acute phase of dengue fever, leading to thrombocytopenia [33]. Moreover, platelet consumption due to disseminated intravascular coagulation (DIC), increased apoptosis, and lysis by the complement system are thought to be caused by the DENV infection itself [34].

Cardiac involvement in MIS-C was characterized by abnormal electrocardiogram findings (59%) and elevated Troponin-T and pro-BNP in 68% and 77% of patients, respectively [6, 35, 36]. These findings align with our studies, which showed that all children (100%) with MIS-C displayed cardiac abnormalities, specifically dilatation of the coronary artery, myocarditis, and pericardial effusion. Due to its immune pathophysiology, high-dose IVIG and glucocorticoids are recommended for MIS-C and have been proven to lower the risk of death, ventilation support, and the cardiovascular dysfunction [15, 19, 35, 37].

An excessive immune response and vascular permeability can increase microthrombi and fibrin formation, causing a marked elevation of D-dimer levels. Some MIS-C patients have coagulopathy due to thrombosis, while severe dengue is accompanied by bleeding due to increased D-dimer levels [15, 35]. D-dimer and ferritin are generally high in MIS-C patients and vary throughout hospitalization [6]. Our study revealed a significant difference in D-dimer levels between the two groups (p value = 0.025, 95% CI).

In this study, the AST level was greater in severe dengue patients, and only half of MIS-C patients had increased AST and ALT levels. Albumin levels were found to decrease in both children with severe dengue and those with MIS-C children. COVID-19 infection can cause liver damage via direct injury, excessive proliferation of liver cells from bile duct epithelial cells, and low oxygen levels that disrupt the liver’s oxygen supply and demand [31, 38]. Hence, 63–97% of COVID-19 patients have elevated AST and ALT levels [31]. Although rare, CT or MRI images obtained from MIS-C patients may reveal hepatosplenomegaly [35]. In contrast, increased AST and ALT levels, and hepatomegaly in dengue patients are only observed in severe disease patients. Table 4 summarizes the differences between several laboratory parameters in patients with COVID-19, dengue infection and severe dengue, as proposed by the author.

**Table 4:** Different parameters levels in COVID-19 patients and dengue infection patients

Parameter	COVID-19	Dengue infection	Severe dengue
AST	↑	Normal	↑↑
ALT	↑	Normal/↑	• ↑↑ • AST/ALT ratio > 2
Albumin	↓	Normal	↓
LDH	↑	↑	↑↑
PT	Normal/↑	Normal/↑	↑
aPTT	Normal/↑	Normal/↑	↑
CRP	• ↑ • Severe ↑↑	↑	↑↑
D-dimer	• ↑ • severe ↑↑	↑	↑

*ALT: alanine transaminase; AST: and aspartate transaminase; LDH: Lactate dehydrogenase; PT: Prothrombin Time; aPTT: activated partial thromboplastin time; CRP: C-Reactive protein*

In our study, even severe dengue children with hypotension had normal levels of consciousness. Hepatomegaly was observed in both severe dengue and severe MIS-C patients. Elevated D-dimer levels in severe dengue patients resulted in bleeding, whereas in severe MIS-C, despite a marked increase in D-dimer levels, none manifested as abnormal bleeding. Soon after extensive intravenous fluid therapy for severe dengue, pleural effusion, ascites, and oedema are likely to occur. In cases of severe MIS-C, intravenous resuscitation fluid rarely causes effusion, ascites, or oedema. Table 5 shows the difference between severe dengue and MIS-C based on the abovementioned features, with the terms “wet” and “dry” used after intravenous resuscitation.

**Table 5:** The difference of severe dengue and MIS-C

	Severe Dengue (shock dengue)	MIS-C (Kawasaki-like shock)
**Vital Signs**	- Hypotensive stage: alert, weak - Narrow pulse pressure - Defervescence: temperature drops	- Decrease consciousness - Pulse pressure narrow or wide (depending on the presence of pericardial effusion/heart problems) - Elevated temperature
**Liver**	Hepatomegaly (+)	Hepatomegaly (+/-)
**Elevated D-Dimer**	Resulted in bleeding	Resulted in coagulopathy
**Rash**	In convalescents phase	In initial stage, could easily disappeared
**Soon after intravenous resuscitation fluid**	“WET” - Effusion (+) - Ascites (+) - Edema (+)	“DRY” - Effusion (+) << - Ascites (-/+) - Edema (-/+)

*MIS-C: Multisystem inflammatory syndrome in children*

In dengue, judicious intravenous fluid therapy is crucial, and symptomatic treatment is additional. In MIS-C, high-dose IVIG and glucocorticoids are recommended. Antibiotic administration in cases of severe dengue and MIS-C should be guided by clinical judgement, laboratory parameters, and microbiological data to mitigate the risk of antibiotic resistance. Antimicrobial stewardship remains a viable approach for regulating the unnecessary use of antibiotics [39].

## Conclusion

Both severe MIS-C and severe dengue patients require PICU treatment and surveillance due to the risk of fatality. MIS-C and severe dengue must be distinguished, especially in dengue-endemic countries. Rash and nonpurulent conjunctivitis, and platelet, AST, and D-dimer lever levels can distinguish severe MIS-C from severe dengue. In early management, NS-1 is more critical than dengue IgM and IgG for detecting dengue promptly and initiating treatment.

### Limitations

This study’s execution is subject to some limitations. Each patient underwent various laboratory tests, and the small sample size may have affected the results. More samples and standardized laboratory analyses are needed to strengthen this study.

## Data Availability

The data presented in this study are within the manuscript.
